# Awareness and preparedness level of medical workers for radiation and nuclear emergency response

**DOI:** 10.3389/fpubh.2024.1410722

**Published:** 2024-06-17

**Authors:** Xinyu Xu, Yanjun Xie, Hongqiu Li, Xining Wang, Shaoteng Shi, Zhihao Yang, Yuemin Lan, Jing Han, Yulong Liu

**Affiliations:** ^1^Department of Occupational and Environmental Health, School of Public Health, Health Science Center, Xi’an Jiaotong University, Xi’an, China; ^2^Global Health Institute, Health Science Center, Xi’an Jiaotong University, Xi’an, China; ^3^Department of Oncology and Occupational Disease, The Second Affiliated Hospital of Soochow University, Suzhou, China; ^4^State Key Laboratory of Radiation Medicine and Protection, School of Radiation Medicine and Protection, Medical College of Soochow University, Suzhou, China

**Keywords:** medical workers, radiation, nuclear emergency, awareness, training programs

## Abstract

Radiological science and nuclear technology have made great strides in the twenty-first century, with wide-ranging applications in various fields, including energy, medicine, and industry. However, those developments have been accompanied by the inherent risks of exposure to nuclear radiation, which is a source of concern owing to its potentially adverse effects on human health and safety and which is of particular relevance to medical personnel who may be exposed to certain cancers associated with low-dose radiation in their working environment. While medical radiation workers have seen a decrease in their occupational exposure since the 1950s thanks to improved measures for radiation protection, a concerning lack of understanding and awareness persists among medical professionals regarding these potential hazards and the required safety precautions. This issue is further compounded by insufficient capabilities in emergency response. This highlights the urgent need to strengthen radiation safety education and training to ensure the well-being of medical staff who play a critical role in radiological and nuclear emergencies. This review examines the health hazards of nuclear radiation to healthcare workers and the awareness and willingness and education of healthcare workers on radiation protection, calling for improved training programs and emergency response skills to mitigate the risks of radiation exposure in the occupational environment, providing a catalyst for future enhancement of radiation safety protocols and fostering of a culture of safety in the medical community.

## 1 Introduction

Over the past century, there has been a swift advancement in the field of radiological sciences and nuclear technologies, leading to their extensive utilization in various societal aspects. While reaping the advantages of employing these technologies in energy generation, radiological healthcare, and various sectors, we are also vulnerable to dangers arising from radioactivity and nuclear mishaps (1). The concept of nuclear radiation, which involves particles or electromagnetic waves emitted by radioactive substances, has been a great concern due to its significant impact on human health and safety (2, 3). Moderate to high doses of irradiation can adversely affect tissues (4, 5), and there is growing evidence of stochastic effects at low doses (100 mGy) (6, 7). In the low dose range (8, 9), the dose–response has been positive for some cancers in medical personnel (10, 11). These risks highlight the critical importance of understanding and managing exposure to ionizing radiation in the occupational environment, particularly for medical personnel who are often on the front line of radiological and nuclear emergencies.

Since the 1950s, medical radiation workers have experienced a notable reduction in occupational doses as a result of enhanced measures for radiation protection (12). Although most healthcare workers acknowledge the seriousness of radiation accidents, there seems to be a general lack of in-depth risk awareness, a poor understanding of protective measures, and inadequate emergency response skills (13, 14). Surveys were conducted by Jafri and colleagues to evaluate the level of radiation safety knowledge among employees working with radiation in Karachi, Pakistan’s radiology, nuclear medicine, and radiotherapy centers. The analysis of the survey showed that radiation therapy workers had only 4.9 per cent correct knowledge of dose-to-control-area dose limits and only 10 per cent correct definition of medical exposures (15). In addition, the level of proficiency in technical and practical skills varies widely, with some demonstrating a high level of competence while others struggle with basic procedures. A study from China showed that 43.9% of responders considered on-site epidemiological investigations to be the weakest skill acquired by emergency responders. Additional areas of weakness comprised personal protective measures (25.7%), crisis psychological support (25.6%), successful response to nuclear and radiological incidents (25.6%), and evaluation of hazards (21.4%) (16). There is also an apparent lack of knowledge in standard policy development and structured educational curricula for these unique situations (17). There is a universality of challenges faced by healthcare workers in various countries, such as Japan, the United States and the Netherlands, to varying degrees, including resource constraints, lack of awareness and limited training opportunities. However, there are also disparate issues such as policy and regulatory differences, cultural and language barriers that need to be addressed (18, 19).

The importance of training medical personnel in radiological and nuclear emergencies has waxed and waned, influenced by social events and the political climate. However, the Chornobyl disaster, the Fukushima accident, and, more recently, increasingly complex radiological devices are stark reminders of the urgency and necessity of comprehensive education and preparedness. Knowledge and operational implementation gaps are identified through the analysis of past accident management experiences, aiding in the understanding of essential areas requiring improvement (1). We need to improve experience in addition to theoretical knowledge training. Assurance of ongoing technological advances and extensive research efforts promise to diversify training methods and enhance the emergency preparedness of health-care workers. The aim of this study is to provide a comprehensive assessment of the current state of preparedness and knowledge among healthcare professionals in dealing with radiological and nuclear emergencies by analyzing and assessing information gathered from published sources and input from partners. By identifying specific gaps in perception and competence, we hope to propose targeted measures and references that will contribute to the overall efficiency of the healthcare system in responding to such crises.

## 2 Medical personnel’s awareness of radiation and nuclear exposure risks

### 2.1 Basic concepts of radiation and nuclear exposure (sources and types of radiation and nuclear exposure)

**Figure 1:**
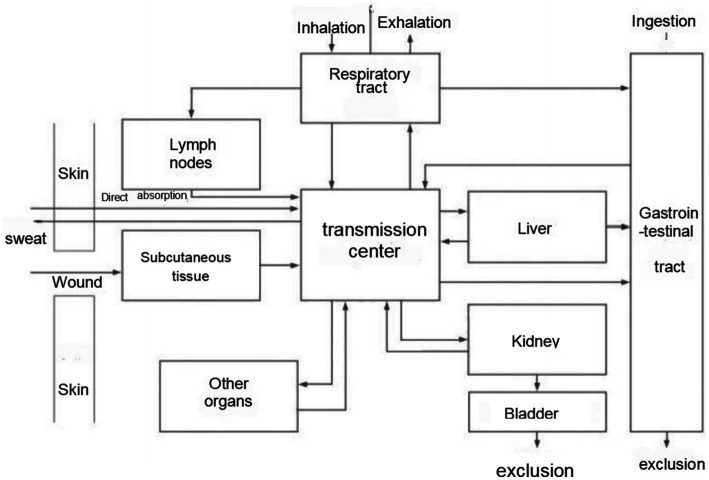
Uptake routes of radionuclides into the body translocation in and excretion from the body.

Ionizing radiation (IR) refers to the particle radiation and electromagnetic radiation that emit energy outward in the form of particles or waves, directly or indirectly promoting the ionization and decomposition of substances, and it has the characteristics of short wavelength, high energy, and high frequency. Nuclear exposure mainly refers to the accidental release of radioactive materials in nuclear facilities (such as nuclear power plant’s radioactive distribution devices), exposing workers and the public to ionizing radiation exceeding or equivalent to the prescribed limits (20, 21). Ionizing radiation has the ability to directly impact biological macromolecules like proteins by breaking down their molecular structure. It can also ionize and excite water molecules, leading to the creation of numerous superoxide anion free radicals, hydroxyl free radicals, and other reactive oxygen free radicals. This indirect effect can result in harm to the hematopoietic system, nervous system, reproductive system, and immune system. Severe cases can lead to disability, cancer, and even death (Figure 1) (22).

Ionizing radiation is usually divided into artificial and natural radiation according to the different radiation sources. Among them, the sources of artificial radiation mainly include the radiation generated by nuclear energy facilities and the application of nuclear technology or nuclear explosions. However, artificial radiation broadly impacts the public, mainly from medical applications (21). In addition, according to international standards (23), radiation doses below 100 mGy are defined as “low-dose radiation,” and radiation workers in medical institutions are the main exposure groups of low-dose occupational nuclear radiation (24). The increasing yearly exposure and participation in radioactive work by institutions and personnel can be attributed to the progress and growth of medical services and nuclear power projects.

### 2.2 Exposure status of medical personnel: changes in occupational exposure dose status and hazards

Ionizing radiation mainly acts on the human body through external and internal irradiation. For medical radiologists, the most common radiation pathway is external irradiation. In order to objectively understand the dose level of medical radiologists and protect the health status of radiation workers, the International Atomic Energy Agency and other international organizations have permanently attached great importance to individual dose monitoring (25). China has also formulated a series of corresponding laws, regulations, standards, and so on, with individual dose monitoring as an essential part of estimating the population exposure dose and preventing it from being exposed to excessive exposure. The Basic Standard for Radiation Source Safety and Ionizing Radiation Protection (GB18871-2002) specifies that the annual practical dose limit for five consecutive years is 20 mSv on average, and the practical dose limit for any 1 year is 50 mSv (26). Monitoring results in China have found that the annual effective dose of most occupationally exposed people is far below the dose limit, Which is most likely due to advances in medical technology that block more radiation (27, 28). A 7-year dose monitoring data of Chinese medical radiologists showed that the average annual effective dose of more than 94.5% of medical radiologists was lower than 1 mSv/a and decreased year by year (29).

Systemic or local radiation damage caused by ionizing radiation on the human body can be divided into acute, subacute, and chronic radiation sicknesses (30). Its influence on human health and its mechanism of action has always been a hot topic in scientific research. Su Yiwei found that being exposed to small doses of ionizing radiation in nuclear power industry employees led to reduced counts of white blood cells, neutrophils, and lymphocytes, in addition to lower levels of hemoglobin, average volume of red blood cells, and average concentration of red blood cell hemoglobin when compared to the control group, suggesting that low dose ionizing radiation has an effect on peripheral blood cells of radiation workers in nuclear power industry and has a certain cumulative damage effect (31). One key area of study is the impact of ionizing radiation on the functionality of the liver and kidneys. The liver is more sensitive to ionizing radiation, and the damage is mainly manifested as radiation hepatitis and fibrosis (32). Alanine aminotransferase (ALT) is a sensitive index of liver damage. In their research, Song Haiyan and colleagues discovered that among 1,646 radiation workers in Nanjing, the incidence of elevated ALT levels was notably greater in the radiation-exposed group compared to the control group (33). However, most of the medical staff have little contact with patients exposed to nuclear radiation, limited treatment experience, and most of them do not have the relevant knowledge and skills to care for and protect nuclear radiation patients and their ability to treat nuclear radiation patients in hospitals needs to be improved (34). At present, most hospitals at home and abroad still lack standardized hospital emergency treatment procedures and nursing technical training guidance for nuclear radiation accidents (35) in order to ensure that nuclear radiation patients receive systematic and professional medical treatment and nursing and reduce the harm caused by nuclear accidents to patients.

### 2.3 Principles of radiation protection

In order to protect both individuals and the environment from the negative effects of ionizing radiation, the safety guidelines set forth by the IAEA establish key principles, requirements, and measures to control human exposure to radiation and the release of radioactive substances into the surrounding areas. This is done to minimize the potential for incidents resulting in the loss of supervision over the nucleus of a nuclear reactor, a chain reaction, a radioactive source, or any other radiation emanating source, and to alleviate the ramifications of such occurrences. The rationality of radiation protection is linked to three key principles: protection optimization and the implementation of dose constraints (36). Table 1 demonstrates guideline values for limiting the exposure of nuclear emergency response personnel. Different standard requirements appropriate to the likelihood and magnitude of exposure are applied to different exposure scenarios, such as life-saving operations, operations in catastrophic situations with significant impacts, and operations to avoid large collective doses. For those healthcare workers who are directly involved with radiation sources, the following protection principles should be the main ones to understand: (a) Main dangers associated with ionizing radiation; (b) Basic dimensions and units for radiation protection; (c) the requirements for radiation protection (including optimized protection and dose limitation); (d) the primary measures of practical radiation protection; (e) task-specific relevant issues; (f) the immediate notification of designated personnel in the case of unforeseen events involving an increased risk of radiation exposure; and (g) the actions that may be required in the case of an accident, if appropriate (38). Table 2 shows the attributes, functions, and precautions of PPE used in different nuclear radiation scenarios.

**Table 1:** Guideline values for limiting exposure of emergency responders.

Task	Guidance value
Life-saving actions	$H_{p}\left(10\right)<500\mathrm{mSv}$ E<500mSv $AD_{T}d<\frac{1}{2}AD_{T}e$
Measures to mitigate extreme deterministic consequences and measures to avert the emergence of disastrous circumstances that could profoundly impact both individuals and the ecosystem.	$H_{p}\left(10\right)<500\mathrm{mSv}$ E<500mSv $AD_{T}d<\frac{1}{2}AD_{T}$ e
Take steps to avoid high-dose collection	$H_{p}\left(10\right)<100\mathrm{mSv}$ E<100mSv $AD_{T}d<0.1AD_{T}e$

$H_{p}\left(10\right)$ , Personal dose equivalent where d = 10 mm; $AD_{T}$ , RBE weighted absorbed dose to a tissue or organ; *E*, Effective dose; ${\mathrm{AD}}_{T}$ d, Values of RBE weighted absorbed dose to a tissue or organ given; ${\mathrm{AD}}_{T}e$ , Values of RBE weighted absorbed dose to a tissue or organ given in table II.1 of GSR Part 7 (37).

IAEA No. GSG-7 Justification of Practices. Including Non-Medical Human Imaging.

**Table 2:** Use of personal protective equipment in different nuclear radiation scenarios.

Types of protective clothing		Characteristics of use
Aprons for protection against penetrating radiation		**Properties**: The apron, which is flexible on both sides, provides protection for the chest and back against radiation scatter that trails behind the body. Its thickness is equivalent to that of 1/3 millimeters of lead. **Function**: Absorbs roughly 90% of low-energy radiation, like scattered X-rays (tens of kilovolts). It is ineffective against the primary X-rays and gamma rays with higher energy levels (over 100 kilovolts), which are frequently used in nuclear medicine, radiotherapy, and industrial settings. **Caution**: Aprons can be cumbersome and may impede efficiency, leading to increased individual exposure if they fail to offer sufficient shielding.
Gloves and other protective gear Penetrating radiation		**Properties**: Contains up to 0.33 mm of lead; excellent protection for extremities when used as localized shielding. **Function**: Low energy scattered X-rays and electrons (beta particles) can penetrate poor shielding against most radiation. **Caution**: Wearing gloves reduces dexterity and can, therefore, result in large hand doses, larger body doses, and prolonged exposures if not used properly.
Respiratory protection equipment	Filtering face piece respirators (FFP)	**Properties**: Filtering facepiece respirators are made entirely or mostly of filtering material that covers the mouth and nose. The facepiece mask is held by straps and nose clips that help complete the seal. **Function**: FFP respirators are designed for primarily protecting against low to moderate hazard particles. **Caution**: FFP respirators have relatively low nominal protection factors, but the highest retention efficiency FFP3 class filters provide adequate protection for low and limited-risk areas.
	Half mask respirators	**Properties**: Flexible half masks are made of rubber or plastic! **Function**: The cartridges are highly absorbent of gases and vapors and provide a secure seal for the subsequent treatment of contaminants.
	Powered air-purifying respirators with masks	**Properties**: Powered Air Purifying Respirators deliver a consistent stream of air to the mask in order to reduce the ingress of polluted air around a mask that is not fully sealed. **Function**: Respirators capture particulate matter, gases, and vapors. Powered respirators employ about thrice the number of filters compared to unpowered respirators. **Note**: In the event of a respirator malfunction, the mask will provide the individual with sufficient opportunity to evacuate the area affected by contamination.

### 2.4 Popularity and acceptability of protective measures for healthcare professionals

Emergency Medical Services (EMS) personnel may face daily radiation exposure, requiring readiness for responding to both natural and man-made incidents involving radioactive substances. After conducting a thorough literature review, it is evident that EMS personnel continue to lack sufficient knowledge and application of protective measures against nuclear radiation. This deficiency persists even with the existence of thorough guidelines provided by reputable organizations such as the International Commission on Radiological Protection (ICRP) and the International Atomic Energy Agency (IAEA). In a study conducted in 2007, findings revealed that 63% of EMS personnel had undergone training related to radiological terrorism, while only 50% had utilized personal protective gear within the past year (39). In the United States during the same year, a study found that just 30.8% of EMS personnel had undergone training for radiological emergencies (40). In 2019, 12 years later, a study of 433 firefighters and EMS personnel showed that many EMS personnel have low knowledge about radiation exposure and precautions. Numerous EMS personnel lacked adequate training on radiation incidents; 25% had not undergone any of the five types of radiation incident training, while the majority had received less than 1 h of training (62). In a 2020 study of 244 nurses in the US, the average score on the 15-item Radiation Measures and Knowledge Assessment (RMEKA) was only 47.17%, which is failing by academic standards. The study also found that many EMS personnel had not been trained in radiation emergency medical management (EMM), the most common form of EMM. There is a lack of training in nuclear emergency response among first responders, specifically in terms of insufficient knowledge of radiation protection, and first responders do not have a full understanding of the basic concepts, types and hazards of radiation. There is a lack of familiarity with emergency response steps and procedures in the event of a nuclear accident, including how to quickly assess and deal with the situation on site. Lack of knowledge of how to properly use and maintain personal protective equipment and lack of guidance on the selection of protective equipment and scenarios for its use. Unfamiliarity with first aid treatments for radiation exposure and contamination, e.g., how to treat radioactive burns and internal contamination. However, participants who had previously taken a radiation emergency medical management course used online resources. It developed a preparedness plan, and they received higher scores (34) in addition to the lack of attention from organizations such as hospitals, which may be related to funding. A study conducted in 2018 found that just 33% of EMS organizations involved had adequate financial resources to offer training in radiological terrorism (41). The research offers valuable perspective on the understanding and awareness of radiation safety protocols among EMS personnel, including radiation technologists, physicists, and radiology practitioners in various hospital environments. Although technological advances, such as the introduction of digital imaging, have helped to reduce radiation doses (42), no technology can completely replace the need for comprehensive education of medical professionals on nuclear radiation protection measures.

## 3 Knowledge of emergency response and willingness to participate

### 3.1 Popularization and assessment of healthcare personnel’s knowledge of emergency response and rescue

Skills and techniques for responding to public health emergencies are prerequisites (16). Radiation incident training improves workers’ ability to respond to radiation events, protects them from workplace radiation exposure, and minimizes the spread of radiation from the source to uncontaminated people, objects, or areas (62). The prevention training aims to improve the self-efficacy and comfort of emergency personnel during radiation incidents. Moreover, training is beneficial for enhancing the willingness of healthcare workers to work. However, research has found that (69) healthcare workers have received little training on radiation incidents; One quarter of individuals have not undergone any of the five varieties of radiation event preparation, whereas a greater number of individuals have undergone 1 h or fewer of training. A 2005 study (43) found that less than 10% of American healthcare workers received training on radiation terrorism. A 2007 study reported (40) that only about one-third of American healthcare workers had received any training on radiation terrorism. In the same year, a study in Canada found (39) that 63% of healthcare workers had received at least some training on radiation terrorism, but only 50% had worn personal protective equipment in the previous year. The level of training among healthcare workers can vary significantly across different countries and regions due to a variety of factors, including differences in healthcare systems, resources, policies, and cultural attitudes toward preparedness and safety. Faced with these challenges, healthcare systems and policy makers must recognize the importance of emergency preparedness training and invest in developing comprehensive, accessible, and sustainable training plans.

### 3.2 The willingness of healthcare workers to participate in emergency response and influencing factors

In emergencies, healthcare workers are the frontline of patient care. Studies indicate that numerous variables influence the willingness of healthcare personnel to engage in initial medical assistance, including limited foundational understanding of radiation emergency protocols, lacking personal safety perceptions, diminished clinical confidence, inability to recognize radiation-induced injuries, and insufficient patient care experience in radiation-related scenarios. Furthermore, concerns regarding personal and familial safety anxiety have been validated as a crucial factor impacting healthcare workers’ willingness to provide aid (44). Research has shown that healthcare workers may not be willing to respond to abnormal emergencies they perceive as dangerous, and nuclear events have the greatest impact on their work willingness, followed closely by radioactive and chemical events (18). Research shows that medical workers exposed to radiation for a long time are more likely to suffer from leukemia, skin cancer, and female breast cancer (45). In addition to inadequate nuclear radiation awareness among healthcare workers, research has found that insufficient funding, inadequate training and drills, and delayed skill updates are the main limiting factors in promoting health emergency skills and technologies (16).

## 4 Current status of skills and technology

### 4.1 Current radiological and nuclear incident skill requirements for healthcare personnel

Part 4 of GSR Part 3, published by the International Atomic Energy Agency (IAEA), states that it is imperative to ensure that the exposure of emergency workers in an emergency does not exceed 50 mSv. Furthermore, a reference level, expressed in terms of residual dose, should be established, typically within the range of 20–100 mSv. The protection strategy should include reducing the residual dose below the reference level to the greatest extent feasible (46). Advanced practice nurses involved in managing nuclear accidents must be knowledgeable about the safe use of radioactive materials while providing patient care. This is particularly important as they may need to treat severely injured patients before decontamination. It is imperative for all nursing personnel to have a comprehensive understanding of the difference between radiation exposure and contamination. When caring for patients who are potentially radioactive or contaminated, medical professionals should consistently adhere to the ALARA principle, which is an acronym for As Low As Reasonably Achievable. Limiting the time spent with patients, maintaining increased distance from radioactive sources and contaminated patients, utilizing proper shielding, and donning appropriate personal protective gear (PPE) can achieve this goal (47). Employees who handle individuals who may be contaminated are required to utilize proper PPE and personal radiation monitoring devices. PPE consists of overalls, helmets, gloves, footwear, face shields, goggles, and impermeable shoe protectors. Additionally, lead containers and tongs should be used to transfer and store highly radioactive foreign objects (48, 49). Lead or lead-equivalent shielding effectively reduces radiation exposure to X-rays and gamma rays. Several types of shielding are used for this purpose, including lead aprons, mobile lead shielding, leaded glass, and lead barriers. During triage, nursing staff must document all assessments and interventions comprehensively and promptly. This information is critical for clinical evaluation and treatment planning (50). After medical stabilization, trained providers are expected to survey patients for radioactive contamination using appropriate equipment and methodology (51). Besides those mentioned above, they must allocate scarce resources, manage medical countermeasures, prevent secondary infections, and provide mental health services (34).

### 4.2 Analysis of the current status of skills and technology of healthcare personnel

Furthermore, many current nursing staff may lack the necessary education and training to effectively handle radiation incidents. Studies indicate that healthcare professionals employed in emergency facilities and Radiation Injury Treatment Network (RITN) medical centers may have insufficient expertise and abilities required to promptly manage radiation incidents (34). Tener Goodwin Veenema and colleagues observed that radiology is not integrated into the curriculum of nursing schools across the United States. This deficiency results in insufficient preparation and education for both present and upcoming nurses in addressing and managing patients and communities in the event of a nuclear or radiological crisis. Nurses, in turn, are an important part of the healthcare and public health response. Many of the current nursing workforce have not been adequately educated and trained to respond and care for patients and communities during a nuclear emergency. As a result, there is a shortage of trained nurses with the necessary skills to respond to a radiological/nuclear event (27). A survey of 59 hospital emergency department staff in a city (of which nurses accounted for 19%) showed that their level of knowledge and clinical skills in dealing with radioactive disasters was low, only 66%. Nurses performed significantly worse than physicians on several aspects of the survey instrument (51). Other studies have also demonstrated nurses’ lack of preparedness and competence to respond to catastrophic events involving radiological devices (52). Nuclear medicine technicians (NMTs) can provide radiological expertise during a public health crisis involving radioactive materials. NMTs have expertise in health physics, radiation biology, radiation safety, decontamination, and patient care, making them a valuable resource. They also possess skills in the safe handling of radioactive materials (53). Medical toxicologists are uniquely qualified to respond to radiation emergencies. They have formal training in radiation medicine and the necessary skills to select and use personal protective equipment, perform decontaminating, identify toxicity, interpret bioassays, administer chelation therapy, and assess and communicate risks (54). Kinugasa and co-authors revealed that more than 6,000 doctors in 35 Japanese cities underwent radiation emergency medical management education and training within a five-year period, improving their knowledge, skills, and confidence in managing radiation-exposed patients (55).

## 5 Status and development of nuclear emergency response training courses

### 5.1 Status and comparison of existing nuclear emergency response training courses

Surveys in existing research indicate a deficiency in emergency preparedness training among many nurses, particularly in systematic training for biochemical or radiation incidents. Specifically, nurses lack confidence in performing emergency actions (56). Investigations conducted by the International Atomic Energy Agency (IAEA) into specific radiation incidents also suggest that inadequate personnel training has resulted in numerous severe consequences that could have been avoided.

However, the current training courses for disaster emergency nursing are notably limited, with most publications originating from the United States (57). This is particularly evident in the curriculum designed for nursing students, where even in the United States, which prioritizes disaster relief education, only short-term courses are available (58–60). Conversely, professional nurses and doctors receive training in major nuclear energy countries. For instance, Japan offers short-term national standard training courses for members of disaster medical response teams and conducts comprehensive nationwide disaster drills annually. Nevertheless, incidents such as ambulance refusal and hospital rejection occurred during the transportation of personnel in the aftermath of the Fukushima nuclear accident (61). US Department of Defense members must undergo federal certification and engage in fixed training and drills at the community level. Through various training modalities such as pre-service training, targeted training, and regular retraining, trainees are equipped with and sustain the capability for nuclear emergency rescue (62). The nuclear emergency profession is absent from China’s most recent edition of the “Catalog of Undergraduate Programs in Regular Higher Education Institutions (2020 Edition).” Consequently, universities nationwide lack standardized specialized courses dedicated to nuclear emergencies. Furthermore, those institutions that offer such courses encounter challenges akin to those in the United States and Japan, including overly simplistic content, outdated teaching methodologies, and inadequate professional faculty (63, 64).

Overall, nuclear emergency education exhibits numerous deficiencies in China and other prominent nuclear nations globally. The knowledge and curriculum frameworks often diverge from the demands of nuclear emergency roles, resulting in a substantial disparity between training content and practical applications. Consequently, in view of the above shortcomings, combined with the professional subdivision of Chinese colleges and universities, it can be considered to set up a subdivided “nuclear emergency medicine” major under the “disaster medicine” major or other similar majors, with theoretical courses and simulated practical courses are designed by the national nuclear emergency management organizations. On the one hand, we can train students, and on the other hand, we can strengthen the experience of nurses, armed police officers and other major nuclear emergency rescue forces. In addition, we can regularly cooperate and exchange information with major foreign nuclear countries, and carry out activities such as joint emergency response exercises, the development of rescue procedures for nuclear leaks, and the standardization of international nuclear curricula, so as to prevent accidents before they occur.

### 5.2 Exploration of nuclear emergency response training courses

Compared to conventional rescue operations, most radiation training programs face significant challenges in using actual radiation sources due to regulatory, administrative, and safety concerns associated with storage, transportation, and utilization. Furthermore, employing radiation exposure for training purposes raises ethical dilemmas. However, without hands-on practice in situations devoid of risk, trainees’ commitment and psychological readiness diminish, significantly compromising training effectiveness (56). Hence, exploring practical and effective hands-on training methods is crucial to offer healthcare workers optimal training environments alongside theoretical knowledge training.

In Sweden, Italo Masiello et al. developed a virtual reality scenario to test the specific technical skills of nuclear power plant personnel through research (65); In China, Haotengfei et al. have created an emergency drill system grounded in three-dimensional simulation via VR (66); Additionally, In South Korea, Dewey Lee (67) developed a VR based exercise system to facilitate radiation emergency exercises for ordinary citizens.

While VR technology has been increasingly employed in nuclear emergency response drills, there are still some limitations. On the one hand, it has been reported in existing studies that some of the students who used virtual technology for learning suffered from discomfort, nausea, and fatigue due to blurred or incorrectly adjusted visuals, and tight headset straps (68, 69); on the other hand, the closer the VR training is to the real-life situation the better the results will be, however, emergencies such as nuclear leaks are not routinely common events, and the on-site information on situations such as a nuclear leak may not be updated for a very In addition, the high cost of virtual reality equipment, the lack of instructors who can provide training, and the possibility that nuclear radiation-related information may be classified and not easily accessible may also make it difficult to apply virtual reality technology to nuclear emergency rescue drills. However, with the gradual upgrading of technology and the more in-depth relevant research in various countries around the world, it is believed that similar training tools will become more and more abundant, and the emergency response quality of medical personnel will become higher and higher.

## 6 Conclusion

The article provides a comprehensive overview of the current status of occupational irradiation and its associated risks, particularly for medical personnel. Although advances in technology and protective measures have generally reduced levels of occupational irradiation, individual healthcare workers may be exposed to higher doses due to the increasing number of radiological procedures. Acute health effects, such as radiation sickness, and long-term consequences, such as cancer and genetic damage, are of concern, with cancer risk being particularly acute.

The article also examines emergency responders’ knowledge and awareness of protective measures, revealing significant gaps in knowledge, particularly regarding decontamination procedures in the event of a radiological incident. Most respondents incorrectly assessed their proximity to nuclear facilities, highlighting the disconnect between perceived and actual risk. In addition, willingness to respond, perceived competence, and personal safety in radiological emergencies showed good internal consistency and reliability, suggesting that medical staff were prepared in these areas. However, there are apparent deficits in basic radiological knowledge, indicating a need for better education and training.

Concerning the current state of skills and technology, many emergency personnel lacked adequate training for radiation incidents. Much medical personnel rarely engage in emergency response activities in a hospital setting and often lack the time to acquire emergency response skills. Even where relevant training has occurred, the inability to conduct effective drills may gradually diminish the knowledge gained, leading to inadequate emergency preparedness and hindering improvement. This contributes to healthcare professionals’ uncertainty about their ability to respond to natural disasters. In this regard, we suggest that Governments should carry out systematic training programs that cover the basics of nuclear physics, radiation protection, nuclear emergency management so as to ensure that all emergency personnel have a solid theoretical foundation. They should also regularly organize nuclear emergency simulation drills to simulate different types of nuclear accident scenarios, so as to enhance the practical ability and adaptability of emergency personnel. On this basis, a regular retraining mechanism should be established to ensure that the knowledge and skills of emergency personnel are up-to-date and that they master the latest technology and emergency handling methods. The government should The government should increase policy support and funding for nuclear emergency training to ensure the smooth implementation and sustainability of the training program, as well as carry out VR and other enrichment training tools to ensure the implementation of the project.

Ongoing technological advances and extensive research efforts worldwide promise to diversify training methods and enhance the emergency preparedness of healthcare workers. Improving radiation safety and emergency preparedness is a complex and multilayered task involving technology, management, education, policy and other aspects, and in the future, with technological innovation and application, virtual reality (VR) and augmented reality (AR) technology will be utilized for scenario simulation to improve the real-world capabilities of emergency responders. Develop highly sensitive and fast-responding radiation sensors that can monitor radiation levels in real time and provide accurate data. Incorporate knowledge of radiation safety into the school education system to cultivate students’ safety awareness from an early age. This is essential to ensure the health and safety of healthcare workers and the effective management of radiation and nuclear accidents.

## Author contributions

XX: Conceptualization, Project administration, Writing – original draft. YX: Investigation, Resources, Writing – original draft. HL: Investigation, Project administration, Writing – original draft. XW: Software, Supervision, Writing – original draft. SS: Investigation, Validation, Writing – original draft. ZY: Investigation, Validation, Writing – original draft. YLa: Conceptualization, Investigation, Writing – original draft. JH: Resources, Supervision, Writing – review & editing. YLi: Funding acquisition, Investigation, Writing – review & editing.
